# Plant and algal chlorophyll synthases function in *Synechocystis* and interact with the YidC/Alb3 membrane insertase

**DOI:** 10.1002/1873-3468.13222

**Published:** 2018-09-06

**Authors:** Matthew S. Proctor, Jack W. Chidgey, Mahendra K. Shukla, Philip J. Jackson, Roman Sobotka, C. Neil Hunter, Andrew Hitchcock

**Affiliations:** ^1^ Department of Molecular Biology and Biotechnology University of Sheffield UK; ^2^ Institute of Microbiology Czech Academy of Sciences Center Algatech Třeboň Czech Republic; ^3^ Faculty of Science University of South Bohemia České Budějovice Czech Republic; ^4^ Department of Chemical and Biological Engineering ChELSI Institute University of Sheffield UK

**Keywords:** *Arabidopsis*, chlorophyll, chlorophyll synthase, cyanobacteria, high light‐inducible proteins, YidC/Alb3/OxaI

## Abstract

In the model cyanobacterium *Synechocystis* sp. PCC 6803, the terminal enzyme of chlorophyll biosynthesis, chlorophyll synthase (ChlG), forms a complex with high light‐inducible proteins, the photosystem II assembly factor Ycf39 and the YidC/Alb3/OxaI membrane insertase, co‐ordinating chlorophyll delivery with cotranslational insertion of nascent photosystem polypeptides into the membrane. To gain insight into the ubiquity of this assembly complex in higher photosynthetic organisms, we produced functional foreign chlorophyll synthases in a cyanobacterial host. Synthesis of algal and plant chlorophyll synthases allowed deletion of the otherwise essential native cyanobacterial gene. Analysis of purified protein complexes shows that the interaction with YidC is maintained for both eukaryotic enzymes, indicating that a ChlG‐YidC/Alb3 complex may be evolutionarily conserved in algae and plants.

## **Abbreviations**

**β‐DDM**, n‐dodecyl‐β‐D‐maltoside

**ChlG**, chlorophyll synthase

**HliD**, high light‐inducible protein D

**WT**, wild‐type

Chlorophyll is the major light‐harvesting pigment in plants, algae and cyanobacteria. Solar energy is absorbed by chlorophyll situated in membrane intrinsic photosystems and used for charge separation, which drives ATP and NADPH production. The structures of these photosystems show chlorophyll molecules in specific arrangements allowing highly efficient light capture and energy transfer 1, 2, 3, 4. The complexity and hydrophobicity of these protein–pigment complexes means that their assembly is dependent on numerous auxiliary proteins and that the insertion of chlorophyll and other cofactors must be co‐ordinated with the assembly process 5, 6.

**Figure 1:**
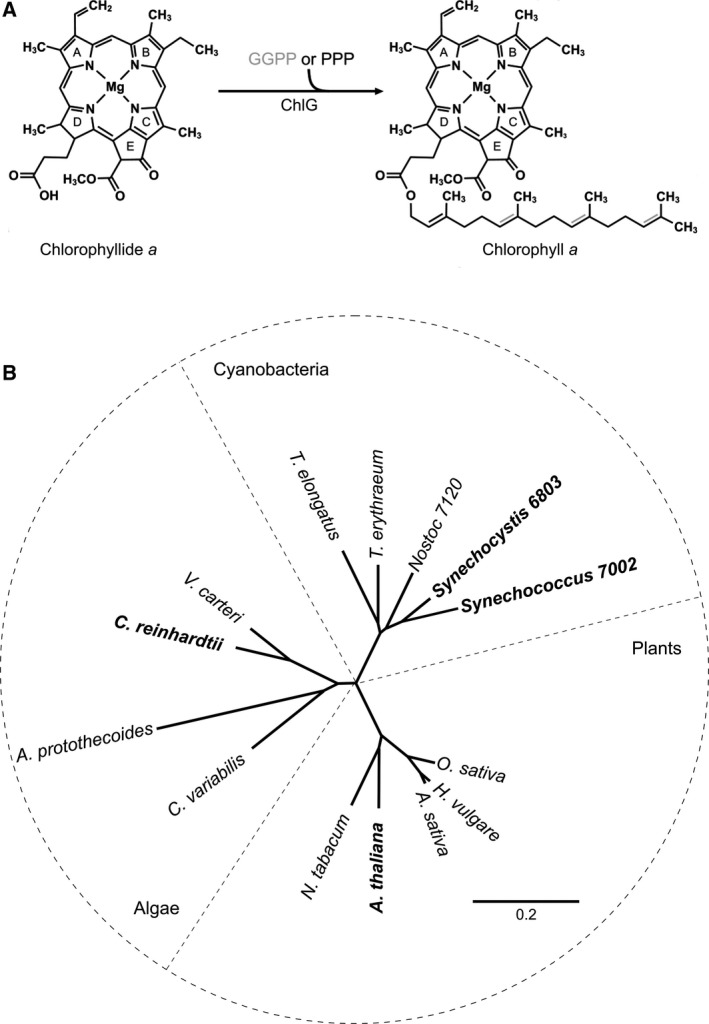
The reaction catalysed by ChlG and protein phylogeny of enzymes from different phototrophic organisms. (A) ChlG catalyses the esterification of chlorophyllide with either GGPP or PPP resulting in GG‐chlorophyll *a* or phytylated chlorophyll *a*. Three carbon–carbon double bonds (shown with grey lines) in the geranylgeranyl tail of GG‐chlorophyll *a* are sequentially reduced to phytol by the geranylgeranyl reductase ChlP. (B) Protein phylogeny of chlorophyll synthases from representative cyanobacteria, algae and plants. The chlorophyll synthases used in this study are shown in bold. The scale bar indicates the number of amino acid substitutions per site.

Chlorophyll synthase (ChlG) is the terminal enzyme of the chlorophyll biosynthesis pathway, catalysing the esterification reaction that adds a tetraprenyl tail to the propionate residue at the C17 position on ring D of chlorophyllide (Fig. 1A). The macrocycle can be esterified with either geranylgeranyl from geranylgeranyl pyrophosphate (GGPP) or phytol from phytyl pyrophosphate (PPP); in the former case the tail is subsequently reduced to phytol by the geranylgeranyl diphosphate reductase, ChlP. In a previous study, Chidgey *et al*. 7 investigated handover of chlorophyll from ChlG to nascent light‐harvesting polypeptides in the model cyanobacterium *Synechocystis* sp. PCC 6803 (hereafter *Synechocystis*). Immunoprecipitation using FLAG‐tagged ChlG retrieved an enzymatically active protein–pigment complex containing the high light‐inducible protein HliD (Ssr1789), the photosystem II (PSII) assembly factor Ycf39 (Slr0399) and the membrane insertase YidC (Slr1471), as well as chlorophyll, the immediate chlorophyll precursor chlorophyllide and the carotenoids zeaxanthin, β‐carotene and myxoxanthophyll. The pigment binding capabilities of the complex were attributed to the ChlG‐HliD ‘core complex’ 7. HliD belongs to a conserved family of high‐light‐induced proteins (Hlips) 8, members of which share significant sequence similarity with plant chlorophyll *a*/*b*‐binding proteins and possess a conserved chlorophyll‐binding motif 9. Stavleva *et al*. 10 found that HliD binds chlorophyll *a* and β‐carotene in a 3 : 1 ratio; the β‐carotene can quench excited chlorophylls 11, suggesting that HliD has a photoprotective function. The additional carotenoids, zeaxanthin and myxoxanthophyll, may bind at the ChlG/HliD interface 11.

The two other major components of the complex are Ycf39, an atypical short‐chain dehydrogenase with an unknown role in PSII assembly 12, 13, and YidC, which belongs to the evolutionarily conserved YidC/Alb3/OxaI family of membrane insertase proteins found in bacteria, mitochondria and chloroplasts. YidC/Alb3/OxaI have a role in the folding and partitioning of transmembrane polypeptides into the phospholipid bilayer 14, 15. Thylakoid membrane biogenesis in cyanobacteria and higher photosynthetic organisms is known to be dependent on YidC/Alb3 16, 17, and the study by Chidgey *et al*. 7 established a link between chlorophyll biosynthesis and YidC‐dependent cotranslational insertion of nascent light‐harvesting polypeptides into membranes.

The high degree of similarity between cyanobacterial photosystems and those of higher photosynthetic organisms raises the possibility of conserved chlorophyll handover systems in the chloroplasts of algae and plants, investigated here by expressing foreign ChlG genes in the model cyanobacterium *Synechocystis*. We demonstrate that under typical laboratory growth conditions the production of the algal and plant chlorophyll synthases allows deletion of the essential native *chlG* without obvious phenotypic consequences. Immunoprecipitations using the tagged heterologous synthases revealed that the ChlG‐YidC complex is maintained in both cases, but that the HliD and Ycf39 components do not interact with the eukaryotic enzymes, which also do not copurify with bound pigments. We additionally show that Ycf39 is lost from the cyanobacterial complex following high light stress, consistent with its proposed role in chlorophyll recycling under photo‐damaging conditions.

## Materials and methods

### Bioinformatics

Sequence alignments were performed using ClustalW 18 and the phylogenetic tree was generated in Geneious version 10.0.2 (http://www.geneious.com; 19). NCBI accession numbers of proteins used for both analyses are provided in Table S1.

### Growth conditions

*Synechocystis* strains were grown at 30 °C in a rotary shaker with moderate light (30–50 μmol photons·m^−2^·s^−1^) in BG11 medium 20 plus 10 mm TES (Sigma‐Aldrich, Dorset, UK)‐KOH pH 8.2. For growth on plates, 1.5% (w/v) agar and 0.3% (w/v) sodium thiosulfate were added. Photoheterotrophic growth medium contained 5 mm glucose. Zeocin (2.5–20 μg·mL^−1^) and kanamycin (5–40 μg·mL^−1^) were included where appropriate. For purification of protein complexes, cultures were grown photoautotrophically with ~ 100 μmol photons·m^−2^·s^−1^ illumination in 8 L vessels bubbled with sterile air and mixed using a magnetic stirrer. To perform light shock experiments, 8 L cultures were grown with 40 μmol photons·m^−2^·s^−1^ to log phase (optical density at 750 nm (OD_750_) ≈ 0.7) and 4 L was harvested as a moderate light control. The remaining 4 L was diluted twofold with fresh media to reduce cell shading and irradiated at high light (∼800 μmol photons·m^−2^·s^−1^) for 80 min. The 8 L cultures were maintained at 30 °C using a temperature coil connected to a thermostat‐controlled circulating water bath.

### Construction of *Synechocystis* strains

See Table 1 for strains of *Synechocystis* used in this study and Tables S2–S3 for plasmid and primer details. *Escherichia coli* JM109 competent cells (Promega UK, Southampton, UK) were used for cloning. The *Arabidopsis thaliana* (AT3G51820; NCBI accession: AY081481) and *Chlamydomonas reinhardtii* (CHLREDRAFT_5437; NCBI accession: XP_001701588) *chlG* genes lacking the sequence coding for the N‐terminal chloroplast transit peptides (57 or 43 amino acids respectively, according to the ChloroP 1.1 Server 21) were synthesized with codons optimized for expression in *Synechocystis* (Integrated DNA Technologies, Coralville, IA, USA). The *chlG* gene from *Synechococcus* sp. PCC 7002 (SYNPCC7002_A0548; NCBI accession: ACA98555) was amplified from genomic DNA using Q5 DNA Polymerase (New England Biolabs UK, Hitchin, UK) and primers 7002_G_F and 7002_G_R. The *Rhodobacter (Rba.) sphaeroides* 2.4.1 *bchG* gene (Rsp_0279; NCBI accession: 3719288) was synthesized with codons optimized for expression in *Synechocystis* (Integrated DNA Technologies). The genes were digested and cloned into the *Not*I and *Bgl*II sites of the plasmid pPD‐*N*FLAG such that they were in frame with an N‐terminal 3xFLAG tag; following homologous recombination into the *Synechocystis* genome, the tagged construct replaced the *psbAII* gene so expression is under the control of the *psbAII* promoter 22. The pPD‐*N*FLAG‐*chlG* plasmid described in 7 was used to replace the *psbAII* gene with the FLAG‐tagged *Synechocystis chlG* gene.

**Table 1:** Strains of *Synechocystis* sp. PCC 6803 used in this study

Strain	Abbreviation	Properties	**Reference/source**
Wild‐type	WT	Glucose‐tolerant WT strain of *Synechocystis* sp. PCC 6803	44
*psbAII*::FLAG‐6803_*chlG*	–	N‐terminally FLAG‐tagged copy of *Synechocystis chlG* in place of *psbAII* gene, kanamycin resistant (kan^R^)	This study
*psbAII*::FLAG‐6803_*chlG*/Δ*chlG*	FLAG‐6803	*psbAII*::FLAG‐6803_*chlG* strain in which native *chlG* gene is replaced with a zeocin resistance (zeo^R^) cassette, kan^R^	This study
*psbAII*::FLAG‐7002_*chlG*	–	N‐terminally FLAG‐tagged copy of *Synechococcus* sp. PCC 7002 *chlG* in place of *psbAII* gene, kan^R^	This study
*psbAII::FLAG‐*7002_*chlG*/Δ*chlG*	FLAG‐7002	*psbAII*::FLAG‐7002_*chlG* strain in which the native *chlG* gene has been deleted, kan^R^, zeo^R^	This study
*psbAII*::FLAG‐Cr_*chlG*	–	N‐terminally FLAG‐tagged copy of *Chlamydomonas reinhardtii chlG* in place of *psbAII* gene, kan^R^	This study
*psbAII*::FLAG‐Cr_*chlG*/Δ*chlG*	FLAG‐Cr	*psbAII*::FLAG‐Cr_*chlG* strain in which the native *chlG* gene has been deleted, kan^R^, zeo^R^	This study
*psbAII*::FLAG‐At‐*chlG*	–	N‐terminally FLAG‐tagged copy of *Arabidopsis thaliana chlG* in place of *psbAII* gene, kan^R^	This study
*psbAII*::FLAG‐At‐chlG/Δ*chlG*	FLAG‐At	*psbAII*::FLAG‐At‐*chlG* strain in which the native *chlG* gene has been deleted, kan^R^, zeo^R^	This study
*psbAII::*FLAG‐*bchG*	–	N‐terminally FLAG‐tagged copy of *Rhodobacter sphaeroides bchG* in place of *psbAII* gene, kan^R^	This study
*psbAII::*FLAG‐*bchG*/Δ*chlG* ^*NS*^	–	*psbAII*::FLAG‐*bchG* strain with nonsegregated deletion of the native *chlG* gene, kan^R^, zeo^R^	This study
Δ*psbB*	–	*psbB* (slr0906) deletion strain, zeo^R^. Cannot grow under photoautotrophic conditions	45

The sequence‐verified (GATC Biotech, Konstanz, Germany) plasmids were introduced into *Synechocystis*, with transformants selected with 5 μg·mL^−1^ kanamycin and genome copies fully segregated by restreaking on plates with sequentially increased antibiotic up to a concentration of 40 μg·mL^−1^. The native *Synechocystis chlG* gene (slr0056) was subsequently deleted from the strains containing the tagged genes by replacement of part of the gene with a zeocin resistance cassette using the linear mutagenesis construct described by Chidgey *et al*. 7. Segregation at the *psbAII* and *chlG* loci was confirmed by PCR with oligonucleotides AH47/AH48 and AH102/AH103, respectively. The sequence of each foreign *chlG* gene was confirmed to be correct and in frame with the FLAG‐tag by PCR amplification from the genome.

### UV‐visible absorbance spectroscopy

The UV‐visible absorbance spectra of cells, membranes and protein complexes were measured in a Cary 60 UV‐Vis spectrophotometer (Agilent Technologies LDA UK Ltd, Stockport, UK) at room temperature with appropriate media/buffer baseline correction.

### Pigment analysis

Pigments were separated by reverse‐phase HPLC on an Agilent 1200 HPLC system using a Discovery^®^ HS C18 5 μm column (column dimensions: 25 cm × 4.6 mm) according to a method slightly modified from 23. Pigments were extracted in methanol at room temperature and applied to the column equilibrated in acetonitrile/water/trimethylamine (9 : 1 : 0.01, v/v/v). After 2 min a linear gradient of 0–100% ethyl acetate was applied over 15 min followed by isocratic ethyl acetate for 5 min at a flow rate of 1 mL·min^−1^ at 40 °C. Absorbance was monitored at 450 and 665 nm and chlorophyll and carotenoid species were identified by their absorption spectra and retention time.

### Quantification of chlorophyll and chlorophyll precursors

Chlorophyll content was determined spectrophotometrically following extraction from cell pellets (from 1 mL of culture at OD_750_ ≈ 0.4) with 100% methanol according to 24. To assess the level of chlorophyll precursors, pigments were extracted from cell pellets (from 2 mL of OD_750_ ≈ 0.4) and analysed by HPLC with two fluorescence detectors as described by Pilný *et al*. 25.

### Membrane preparation, solubilisation and anti‐FLAG immunoprecipitation

*Synechocystis* cells expressing FLAG‐tagged ChlG were grown to an OD_750_ of ≈ 0.7 and harvested by centrifugation (17 700 ***g***, 4 °C, 20 min). The subsequent procedures were performed either in the dark or under dim green light. Pellets (from 4 L of culture) were washed and resuspended in FLAG‐buffer (25 mm sodium phosphate pH 7.4, 10 mm MgCl_2_ and 50 mm NaCl, 10% (w/v) glycerol and EDTA‐free Protease Inhibitor (Roche, West Sussex, UK)), mixed with an equal volume of 0.1 mm glass beads (BioSpec, Bartlesville, OK, USA) and broken in a Mini‐Beadbeater‐16 (BioSpec). Soluble and membrane proteins were separated by centrifugation (48 400 ***g***, 4 °C, 30 min) and the membrane fraction was resuspended in 10 mL FLAG‐buffer with 2% (w/v) n‐dodecyl‐β‐D‐maltoside (β‐DDM; Anatrace) and solubilised at 4 °C for 1 h with gentle agitation. Insoluble material was pelleted (48 400 ***g***, 4 °C, 30 min) and the supernatant was diluted twofold in FLAG‐buffer and applied to a 0.3 mL anti‐FLAG‐M2 agarose (Sigma‐Aldrich) column equilibrated in FLAG‐buffer with 0.04% (w/v) β‐DDM (wash buffer). The resin was washed with 20 resin volumes of wash buffer to remove contaminating proteins and FLAG‐tagged proteins were eluted in 400 μL of the same buffer containing 187.5 μg·mL^−1^ 3xFLAG peptide (Sigma‐Aldrich).

### SDS/PAGE and immunodetection

Membrane preparations and FLAG eluates were separated by SDS/PAGE on Invitrogen 12% Bis–Tris NuPage gels (Thermo Fisher Scientific, Loughborough, UK) and stained with Coomassie Brilliant Blue (Bio‐Rad, Watford, UK) or transferred onto polyvinylidene fluoride membranes (Thermo Fisher Scientific) for immunodetection. Membranes were incubated with specific primary antibodies against the 3xFLAG tag (Sigma‐Aldrich), HliD (Agrisera, Vännäs, Sweden), Ycf39 7 and YidC (provided by Jörg Nickelsen, Ludwig‐Maximilians‐University, Munich, Germany) followed by an appropriate secondary antibody (anti‐rat for 3xFLAG, anti‐rabbit for the other primary antibodies) conjugated with horseradish peroxidase (Sigma‐Aldrich) to allow detection using the WESTAR ETA C 2.0 chemiluminescent substrate (Cyanagen, Bologna, Italy) with an Amersham Imager 600 (GE Healthcare, Amersham, UK). For native electrophoresis, isolated complexes were separated on a 4–12% clear native gel exactly as described previously 7.

### Gel filtration chromatography

The eluate from the FLAG column was adjusted to a β‐DDM concentration of 1% (w/v) prior to fractionation using a Bio‐Sep 3000 gel filtration column (Phenomenex, Macclesfield, UK) on an Agilent‐1200 HPLC system, as described in 7.

### Nanoflow liquid chromatography tandem mass spectrometry (LC‐MS/MS)

The FLAG‐eluted proteins were digested with trypsin and analysed by nanoflow LC‐MS/MS as described previously 22.

## Results

### Algal and plant chlorophyll synthases are functional in *Synechocystis*

**Figure 2:**
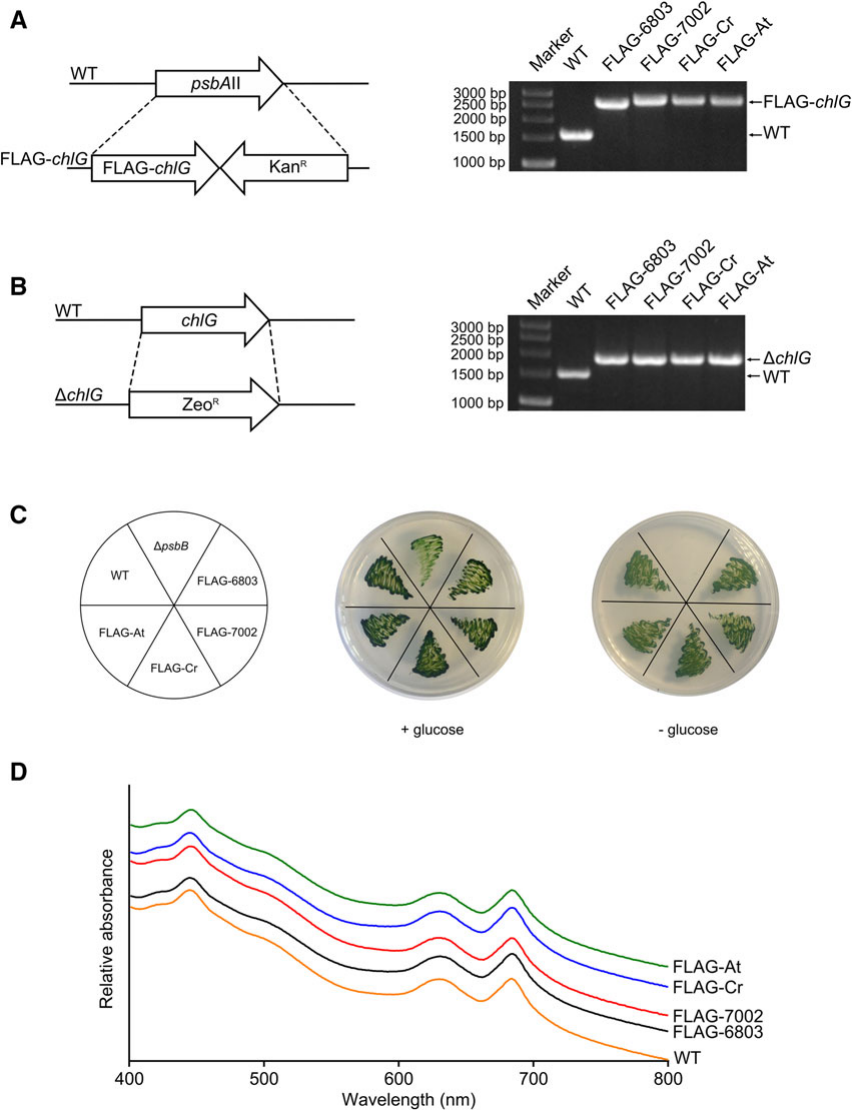
Generation of *Synechocystis* strains expressing foreign *ChlG* genes. (A) Genes encoding 3xFLAG‐tagged ChlG from the organisms indicated were inserted in place of the *psbAII* gene in the *Synechocystis* genome. (B) The native *chlG* gene was subsequently deleted from the strains expressing the foreign genes by replacement with a zeocin resistance cassette. (C) Strains producing foreign chlorophyll synthases that lack the native enzyme grow under photohetrotrophic and photoautotrophic conditions. A Δ*psbB* mutant that cannot grow under photoautotrophic conditions 44 is included as a control. (D) Whole‐cell absorbance spectra of WT and FLAG‐ChlG strains. Spectra are normalized to 575 nm and offset to allow individual traces to be distinguished.

To investigate whether ChlG proteins from algae and higher plants are functional in a cyanobacterial system, and if interactions between foreign ChlGs and the cyanobacterial HliD, Ycf39 and YidC proteins are maintained, a collection of mutant strains was generated in which foreign *chlG* genes encoding N‐terminally 3xFLAG‐tagged proteins were added *in trans* to *Synechocystis* (Table 1). Protein phylogeny shows that the cyanobacterial, algal and plant chlorophyll synthases form separate branches (Fig. 1B), thus enzymes from another cyanobacterium, *Synechococcus* sp. 7002; the model green alga, *Chlamydomonas reinhardtii*; and the model plant species, *Arabidopsis thaliana*, were chosen. After introduction of foreign *chlG* genes at the *psbAII* locus (Fig. 2A), the native *chlG* gene was replaced with an antibiotic resistance cassette (Fig. 2B). Chlorophyll synthase is essential in *Synechocystis*, so complete deletion of the native *chlG* gene is only possible if the introduced foreign gene is active; in a control using the bacteriochlorophllide‐specific bacteriochlorophyll synthase (BchG) from *Rba. sphaeroides*, which cannot esterify chlorophyllide 26, subsequent deletion of the native *chlG* is not possible (Fig. S1).

The resulting strains, *psbAII*::3xFLAG‐7002_*chlG*/Δ*chlG* (FLAG‐7002), *psbAII*::3xFLAG‐Cr_*chlG*/Δ*chlG* (FLAG‐Cr), *psbAII*::3xFLAG‐At_*chlG*/Δ*chlG* (FLAG‐At) are capable of photoheterotrophic and photoautotrophic growth (Fig. 2C) and have similar absorption profiles to the wild‐type (WT) and *psbAII*::3xFLAG‐6803_*chlG*/Δ*chlG* (FLAG‐6803) strains (Fig. 2D), indicating that the foreign ChlG proteins are capable of complementing the deletion of the endogenous enzyme under our standard growth conditions. The chlorophyll content and levels of chlorophyll precursors (Table S4 and Fig. S2) in each of the FLAG‐tagged ChlG strains were also very similar; pertinently no significant increase in chlorophyllide, the substrate of ChlG, was observed in any strain.

### The algal and plant chlorophyll synthases copurify with YidC but not HliD or Ycf39

Solubilised membrane fractions obtained from the FLAG‐6803, FLAG‐7002, FLAG‐Cr and FLAG‐At strains were used for FLAG‐immunoprecipitation experiments. Immunoprecipitations were performed in biological triplicate with consistent results (see the next section for discussion of the unique exception of Ycf39 in FLAG‐7002); for clarity, data from a single representative experiment is presented for each strain. A control immunoprecipitation was carried out using WT *Synechocystis* membranes. None of the interaction partners were observable by immunoblot in the resulting eluate despite a 20‐fold higher loading of the gel; the inability to detect HliD, YidC or Ycf39 rules out nonspecific binding to the anti‐FLAG resin (Fig. S3). Immunoblots of the solubilised membranes from the FLAG‐6803 strain and the flow‐through that did not bind to the anti‐FLAG column confirm that we captured all the FLAG‐tagged bait protein (Fig. S4). The three partner proteins were all present in the flow through but in reduced amounts, consistent with some proportion of each being copurified with the FLAG‐tagged bait protein.

**Figure 3:**
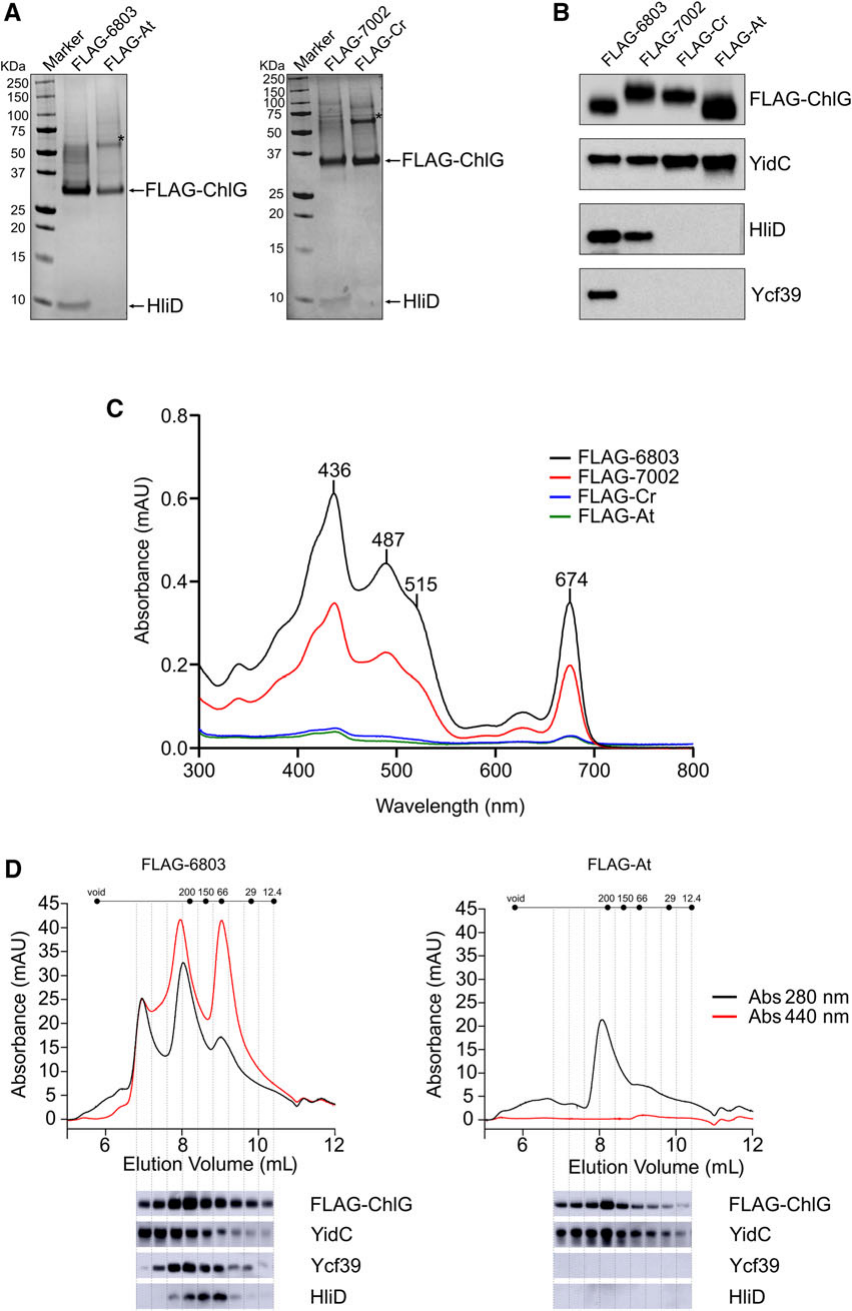
Purification of FLAG‐ChlG from *Synechocystis* strains and identification of interacting proteins. (A) FLAG‐immunoprecipitation eluates were separated by SDS/PAGE and analysed by staining with Coomassie Brilliant Blue. (B) Immunodetection of FLAG‐ChlG, YidC, HliD, and Ycf39 in FLAG‐immunoprecipitation eluates. In (A,B), data from a single experiment are presented but are representative of at least three biological replicates, with the exception of the 7002‐ChlG interaction with Ycf39 (see text and Fig. S8 for further explanation). The asterisk (*) in panel (A) indicates a prominent protein band in the eukaryotic enzyme eluates that cross reacts with both anti‐FLAG and anti‐ChlG antibodies, indicating it is a ChlG dimer. (C) Absorption spectra of FLAG‐immunoprecipitation eluates. (D) HPLC gel filtration chromatography separation of purified cyanobacterial and plant FLAG‐ChlG eluates. Elution of pigment and protein were monitored at 440 (red line) and 280 (black line) nm, respectively. Immunoblot analyses of the HPLC elution fractions are shown below the traces.

The presence of similar amounts of the tagged bait protein in the immunoprecipitation eluates was confirmed by SDS/PAGE (Fig. 3A) and anti‐FLAG immunoblots (Fig. 3B). The FLAG‐6803 and FLAG‐7002 eluates were both visibly coloured and spectrophotometric analysis shows they contain chlorophyll (436, 674 nm) and carotenoids (487, 515 nm) (Fig. 3C). HPLC profiles of the extracted pigments confirmed the presence of myxoxanthophyll, zeaxanthin, β‐carotene and chlorophyll in approximately the same ratio in each eluate (Fig. S5). Conversely, the FLAG‐Cr and FLAG‐At eluates appeared colourless to the naked eye and contained negligible levels of pigment when analysed spectroscopically (Fig. 3C). Consistent with the pigmentation, HliD is present in the cyanobacterial enzyme eluates, visible both on stained gels and by immunodetection, and is absent from both eukaryotic ChlG immunoprecipitations (Fig. 3A,B). HliD was detected in the membranes of cells producing the eukaryotic enzymes (Fig. S6), ruling out that the absence of HliD in the eukaryotic immunoprecipitations is due to any drastic cellular reduction in the amount of the Hlip. Like HliD, Ycf39 was absent from both eukaryotic enzyme eluates (Fig. 3B), consistent with the hypothesis that interaction of Ycf39 with the complex is mediated by HliD 7, 10. Conversely, YidC was present in all the eluates (Fig. 3B). Although it was not visible on stained gels, clear immunoblot signals in the eukaryotic enzyme eluates indicate that a ChlG‐YidC/Alb3 interaction may be conserved in higher chlorophyll‐producing organisms.

The eluates from the FLAG‐6803 and FLAG‐At coimmunoprecipitations were further analysed by gel filtration chromatography, which showed that the pigment is predominantly associated with HliD containing subcomplexes in the FLAG‐6803 elution (as reported previously 7), and confirmed the absence of HliD, Ycf39 and pigments, but the presence of YidC, in the FLAG‐At complex, which eluted as a single major peak (Fig. 3D). Analysis of the same complexes by clear native (CN)‐PAGE (Fig. S7) shows that the *Arabidopsis* FLAG‐ChlG forms a single complex, predicted to be a dimer, while *Synechocytsis* FLAG‐ChlG forms a number of different sized complexes, consistent with the elution profiles from gel filtration. The lack of high molecular weight complexes and pigments in the FLAG‐At eluate suggests that unlike the *Synechocystis* enzyme, *Arabidopsis* ChlG does not interact with PSI 7.

### The interaction between ChlG and Ycf39 is disrupted by high light

**Figure 4:**
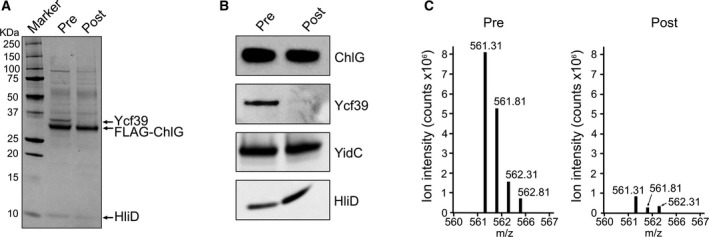
The interaction between ChlG and Ycf39 is abolished by high light. (A) Analysis of pre‐ and postlight‐stressed FLAG‐6803 ChlG immunoprecipitation eluates by SDS/PAGE. (B) Immunoblot analysis of pre‐ and postlight‐stressed FLAG‐6803 ChlG immunoprecipitation eluates confirm the specific loss of Ycf39 following high light stress. (C) Relative intensities of the ‘LAFSEVLASGK’ tryptic peptide representative of Ycf39 as observed by mass spectrometry after tryptic digestion of the eluates.

As described above, all immunoprecipitation experiments were repeated a minimum of three times with independently grown cultures. In all cases the results were consistent, with the sole exception of immunodetection of Ycf39 in the FLAG‐7002 eluates. All three eluates contained pigment and were spectrally very similar (Fig. S8), however, only two of the replicates contained Ycf39 when probed with anti‐Ycf39 antibodies. Every attempt was made to keep the growth conditions identical but we hypothesized that small differences between the biological replicate cultures, such as the light intensity, may be responsible for this variation. In order to investigate this, FLAG‐6803 cells were subjected to high light stress and the pre‐ and postlight stress samples were used in parallel for FLAG‐immunoprecipitations. Analysis of the resultant eluates by SDS/PAGE, immunoblotting and mass spectrometry shows that the light stress treatment specifically results in the loss of Ycf39 from the ChlG complex, while the HliD and YidC interactions are maintained (Fig. 4A–C). Gel filtration of the eluates further demonstrated that the normal light and high light complexes were similar with the exception of the loss of Ycf39 after high light stress (Figs S9–S10). Therefore, Ycf39 dissociates from the ChlG complex when the cells are exposed to high light, presumably as it is recruited by alternative partner proteins involved in the repair of PSII under such conditions of stress. The ability to prepare ChlG‐HliD‐YidC and ChlG‐YidC subcomplexes lacking Ycf39 may be useful for elucidating the specific role of the individual components of the complex in the future.

## Discussion

The processes of chlorophyll biosynthesis and photosystem assembly in phototrophic organisms appear to be co‐ordinated, ensuring efficient channelling of newly produced chlorophyll pigments to *de novo* photosystem polypeptides to enable their cotranslational insertion into the thylakoid membrane and assembly into functioning photosystems 27, 28. Chidgey *et al*. 7 previously identified the ChlG‐HliD‐Ycf39‐YidC assembly complex in the model cyanobacterium *Synechocystis*. To gain insight into whether similar ChlG complexes may form in higher oxygenic phototrophs, FLAG‐tagged algal and plant chlorophyll synthases were heterologously produced in *Synechocystis*; the enzymes replaced the function of the native cyanobacterial enzyme, allowing subsequent deletion of the normally essential endogenous *chlG* gene resulting in strains that had the same growth rate and pigment composition as the WT and a strain producing the FLAG‐tagged native enzyme.

Immunoprecipitations of the FLAG‐tagged ChlG proteins showed that only the most closely related ChlG from *Synechococcus* sp. PCC 7002 eluted with HliD and Ycf39. *Synechococcus* sp. PCC 7002 has close homologues of the *Synechocystis* Ycf39 (SYNPCC7002_A0216) and HliD (SYNPCC7002_A0858) and it is very likely that the same ChlG‐Hlip‐Ycf39‐YidC complex is conserved in this and other related cyanobacteria. Conversely, HliD and Ycf39 were absent from the plant and algal complexes, indicating that the eukaryotic chlorophyll synthases do not interact with these cyanobacterial proteins.

HliD binds β‐carotene and chlorophyll *a* allowing the dissipation of absorbed light energy by chlorophyll to β‐carotene energy transfer 5, 10, consistent with the proposed role of Hlips in photoprotection of chlorophyll‐binding proteins 29, 30, 31. It is possible that similar interactions with Hlip‐like proteins occur in algae and plants 32. *Arabidopsis* one‐helix protein 2 (OHP2), which shows sequence similarity to HliD, associates with YCF244, a homologue of cyanobacterial Ycf39 33. There is evidence for a functional similarity between OHP2 and HliD; an OHP2‐YCF244 complex associates with the PSII intermediate RCII 33 essentially as described for the HliD‐Ycf39 complex in cyanobacteria 13. Light harvesting‐like 3 protein (LIL3) is another Hlip‐like protein that binds pigments and interacts with chlorophyll biosynthesis enzymes (ChlP and protochlorophyllide oxidoreductase) 34, 35, 36, although Hey *et al*. 36 did not find a LIL3–ChlG interaction in *Arabidopsis*.

Ycf39 forms a subcomplex with HliD that appears to associate either with ChlG or the early PSII assembly intermediate RCII 13. The discovery of the ChlG‐HliD‐Ycf39‐YidC complex led to the hypothesis that ChlG associated with HliD and Ycf39 binds the PSII core subunit D1 precursor protein (pD1) as it is being cotranslationally inserted into the membrane by YidC, during which time chlorophyll provided by ChlG can be bound to the polypeptide 7, 13. Consistent with this, the *Arabidopsis* Ycf39 homologue HCF244 is important for translational initiation of *psbA* mRNA 37. The *Synechocystis* strains containing the algal/plant enzymes display no obvious growth or pigmentation phenotype, despite the apparent lack of a ChlG‐HliD/Ycf39 interaction, suggesting that the roles of HliD and Ycf39 within the ChlG complex are not essential to cyanobacteria *in vivo*, at least under the low‐stress conditions used here. This is consistent with the lack of a growth rate or pigment composition defect in *Synechocystis* Δ*hliD* 7 and Δ*ycf39* 13, 38 mutants.

We additionally found that Ycf39 is lost from the FLAG‐6803 ChlG complex under high light stress. Photo‐damaging conditions result in the release of chlorophylls from photosystems; these pigments must be recycled back to the membrane. A Δ*ycf39* mutant is more sensitive to photoinhibition, and there is evidence that the Ycf39‐Hlip complex has a role in chlorophyll recycling and D1 incorporation into PSII during sudden exposure to high irradiance 13. Whether Ycf39 is lost from the complex under other stress conditions, and the mechanism of its release from the ChlG complex, will require further study.

Unlike HliD and Ycf39, YidC was detected in the immunoprecipitation eluates for all enzymes, including the nonfunctional BchG. In the phototrophic bacterium *Rba. sphaeroides,* the photosystem assembly factor LhaA was found to comigrate in CN‐PAGE with the integral membrane protease FtsH, BchG and YidC 39, so (bacterio)chlorophyll synthase‐YidC associations might be widespread in phototrophs. YidC/Alb3 is a member of the evolutionally conserved protein family of membrane insertases 14, 40 and is essential for thylakoid membrane biogenesis in cyanobacteria, algae and plants 16, 17. The discovery of an association between YidC and ChlG led to the hypothesis that YidC fixes chlorophyll‐binding proteins into a configuration that allows for the insertion of newly synthesized chlorophyll molecules from the neighbouring ChlG 7, 41. Unlike HliD, which is visible on stained gels, YidC is not observable by Coomassie blue staining, indicating it is present in the complex in a less than 1 : 1 ratio with ChlG/HliD; this could be confirmed by determination of molar quantities of FLAG‐ChlG, HID and YidC by mass spectrometry in future studies. Nonetheless, the observed interaction between algal/plant ChlG proteins and cyanobacterial YidC provides evidence that these proteins may form similar interactions with Alb3 in their native organisms, implying that co‐ordinated delivery of chlorophyll to nascent light‐harvesting polypeptides *via* ChlG‐YidC/Alb3 interactions is conserved among photosynthetic organisms. *Arabidopsis thaliana* contains a paralog of Alb3 called Alb4, which is required for chloroplast biogenesis 42 and thylakoid protein targeting 43; it is possible the plant ChlG may also interact with this protein. Coimmunoprecipitations with solubilised *Arabidopsis* or spinach thylakoids will allow the *in vivo* partner proteins of the plant enzyme to be confirmed.

## Author contributions

CNH and AH conceived the project. JWC, RS, CNH and AH supervised the project and designed the experiments. MSP, JWC, MKS, PJJ, RS and AH performed the experiments and analysed the data. MSP, JWC and AH prepared the manuscript, which was edited by RS and CNH.

## Supporting information


**Fig. S1.** Production of the *Rhodobacter sphaeroides* 2.4.1 bacteriochlorophyll synthase (BchG) in *Synechocystis* does not allow full deletion of the native *chlG* gene.
**Fig. S2.** Analysis of chlorophyll precursors from strains used in this study.
**Fig. S3**. SDS/PAGE and immunoblot analysis of control FLAG immunoprecipitations from solubilised WT membranes.
**Fig. S4.** Comparison of solubilised FLAG‐6803 membranes applied to anti‐FLAG resin versus the flow through that did not bind.
**Fig. S5**. Analysis of the pigment content of the cyanobacterial FLAG‐ChlG coimmunoprecipitation eluates.
**Fig. S6**. Immunodetection of YidC, Ycf39 and HliD in solubilised membranes of different strains used in this study.
**Fig. S7.** Immunoprecipitation eluates from FLAG‐6803 and FLAG‐At strains separated by 2D CN/SDS/PAGE and stained by SYPRO Orange.
**Fig. S8**. Absorbance spectra of three independent FLAG‐7002 ChlG immunoprecipitation eluates.
**Fig. S9.** Gel filtration chromatography of the pre‐ and postlight stress eluates.
**Fig. S10.** Relative distribution of the components of the pre‐ and postlight stress eluates separated by gel filtration chromatography.
**Table S1.** NCBI accession numbers of chlorophyll synthases from the indicated species.
**Table S2.** Plasmids used in this study.
**Table S3.** Primers used in this study.
**Table S4.** Chlorophyll content of strains used in this study.
