# Supplementary material for: Plant and algal chlorophyll synthases function in Synechocystis and interact with the YidC/Alb3 membrane insertase
Source: FEBS Lett. 2018 Sep 6;592(18):3062–73. doi: 10.1002/1873-3468.13222 (PMC6175206; doi:10.1002/1873-3468.13222)
Supplement: Supplementary file 1 — Fig. S1. Production of the Rhodobacter sphaeroides 2.4.1 bacteriochlorophyll synthase (BchG) in Synechocystis does not allow full deletion of the native chlG gene. Fig. S2. Analysis of chlorophyll precursors from strains used in this study. Fig. S3. SDS/PAGE and immunoblot analysis of control FLAG immunoprecipitations from solubilised WT membranes. Fig. S4. Comparison of solubilised FLAG‐6803 membranes applied to anti‐FLAG resin versus the flow through that did not bind. Fig. S5. Analysis of the pigment content of the cyanobacterial FLAG‐ChlG coimmunoprecipitation eluates. Fig. S6. Immunodetection of YidC, Ycf39 and HliD in solubilised membranes of different strains used in this study. Fig. S7. Immunoprecipitation eluates from FLAG‐6803 and FLAG‐At strains separated by 2D CN/SDS/PAGE and stained by SYPRO Orange. Fig. S8. Absorbance spectra of three independent FLAG‐7002 ChlG immunoprecipitation eluates. Fig. S9. Gel filtration chromatography of the pre‐ and postlight stress eluates. Fig. S10. Relative distribution of the components of the pre‐ and postlight stress eluates separated by gel filtration chromatography. Table S1. NCBI accession numbers of chlorophyll synthases from the indicated species. Table S2. Plasmids used in this study. Table S3. Primers used in this study. Table S4. Chlorophyll content of strains used in this study. [file FEB2-592-3062-s001.docx]

**Proctor *et al.* Supporting Information (Tables S1-S4 and Figures S1-S10)**

**Table S1. NCBI accession numbers of chlorophyll synthases from the indicated species.**

| **Species** | **NCBI accession number** |
| --- | --- |
| *Synechocystis* sp. PCC 6803 | BAA10281 |
| *Synechococcus* sp. PCC 7002 | ACA98555 |
| *Thermosynechococcus elongatus* BP1 | NP_682329 |
| *Nostoc* sp. PCC 7120 | BAB76179 |
| *Trichodesmium erythraeum* IMS101 | WP_011610670.1 |
| *Chlamydomonas reinhardtii* | XP_001701588 |
| *Chlorella variabilis* | XP_005849286.1 |
| *Volvox carteri* | XP_002949134.1 |
| *Auxenochlorella protothecoides* | XP_011396726.1 |
| *Arabidopsis thaliana* | AY081481 |
| *Hordeum vulgare* | NP_001312258 |
| *Oryza sativa subsp. japonica* | [XP_015637650](https://www.ncbi.nlm.nih.gov/protein/XP_015637650) |
| *Nicotiana tabacum* | NM_001325329.1 |
| *Avena sativa* | XP_015637650 |

**Table S2. Plasmids used in this study.**

| **Plasmid name** | **Properties** | **Reference/source** |
| --- | --- | --- |
| pPD-*N*FLAG | 3хFLAG tag encoding sequence flanked by *psbAII* up- and downstream regions to allow replacement of of *psbAII* in *Synechocystis* sp. PCC 6803 genome with the inserted gene. Kanamycin resistant (kan^R^). | Hollingshead *et al*. 2012 |
| pPD-*N*FLAG-*chlG* | *Synechocystis* sp. PCC 6803 *chlG* gene cloned into *Not*I and *Bgl*II sites of pPD-*N*FLAG so in frame with N-terminal 3хFLAG tag. | Chidgey *et al*. 2014 |
| pPD-*N*FLAG-*chlG*_7002 | *Synechococcus* sp. PCC 7002 *chlG* gene cloned into *Not*I and *Bgl*II sites of pPD-*N*FLAG so in frame with N-terminal 3хFLAG tag. | This study |
| pPD-*N*FLAG-*chlG*_Cr | *Chlamydomonas reinhardtii* *chlG* gene cloned into *Not*I and *Bgl*II sites of pPD-*N*FLAG so in frame with N-terminal 3хFLAG tag. | This study |
| pPD-*N*FLAG-*chlG*_At | *Arabidopsis thaliana* *chlG* gene cloned into *Not*I and *Bgl*II sites of pPD-*N*FLAG so in frame with N-terminal 3хFLAG tag. | This study |
| pPD-*N*FLAG-*bchG*_Rs | *Rhodobacter sphaeroides* *bchG* gene cloned into *Not*I and *Bgl*II sites of pPD-*N*FLAG so in frame with N-terminal 3хFLAG tag. | This study |

| **Primer name** | **Sequence (5'-3')** | **Properties** |
| --- | --- | --- |
| 7002_G_F | GGAATTC**GCGGCCGC***A*CCCAATGACGAGTGGTTTTC | *Not*I site in bold, extra base to keep in frame italicised |
| 7002_G_R | GGAATTC**AGATCT**TTAGGAAATCCCCGCATGGC | *Bgl*II site in bold |
| AH47 | AAACGCCCTCTGTTTACCCA | Screening primers for segregation at *psbAII* locus |
| AH48 | TCAACCCGGTACAGAGCTTC |  |
| AH102 | TGCAAGCAACCCGTTACCA | Screening primers for segregation at *chlG* locus |
| AH103 | CCCTTTAGATTTTAGGACGGCGA |  |

**Table S3. Primers used in this study.**

**Table S4. Chlorophyll content of strains used in this study.**

| **Strain** | **Chlorophyll (µg ml^-1^ OD_750_^-1^)** |
| --- | --- |
| WT | 5.0±0.20 |
| FLAG-6803 | 5.1±0.18 |
| FLAG-7002 | 5.2±0.22 |
| FLAG-Cr | 4.6±0.13 |
| FLAG-At | 4.9±0.26 |

Chlorophyll content was determined as described in Materials and methods. Values are the average of three biological replicates ± the standard deviation.

**
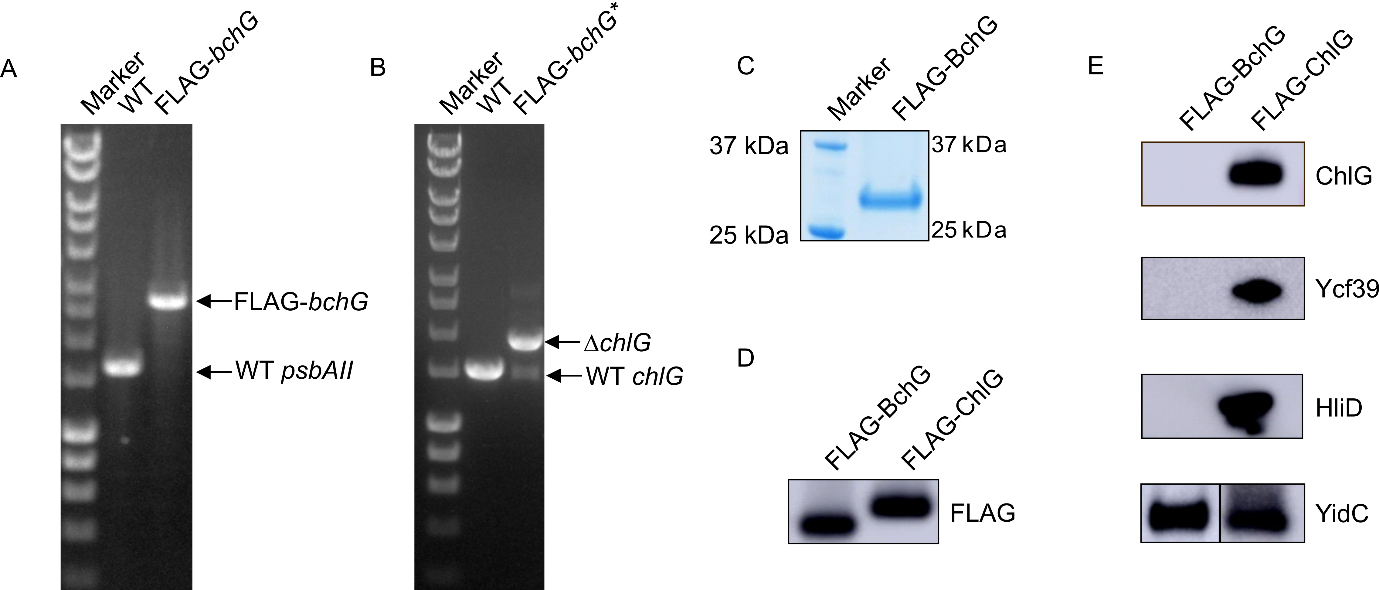
**

**Supplementary Fig. S1**. **Production of the *Rhodobacter sphaeroides* 2.4.1 bacteriochlorophyll synthase (BchG) in *Synechocystis* does not allow full deletion of the native *chlG* gene.** (A) The *Rhodobacter sphaeroides* 2.4.1 *bchG* gene with sequence encoding an N-terminal 3хFLAG tag was inserted at the *psbAII* locus. (B) Subsequent attempts to delete the native *chlG* gene resulted in a non-segregated merodiploid strain containing chromosomes with both WT and mutated copies of *chlG*. (C-D) FLAG-tagged BchG was produced, as confirmed by SDS-PAGE (C) and anti-FLAG immunblots (D) of FLAG-immunoprecipitation eluates. (E) Of the three major interaction partners that co-elute with *Synechocystis* FLAG-ChlG, only YidC was detectable in the FLAG-BchG eluates.

**
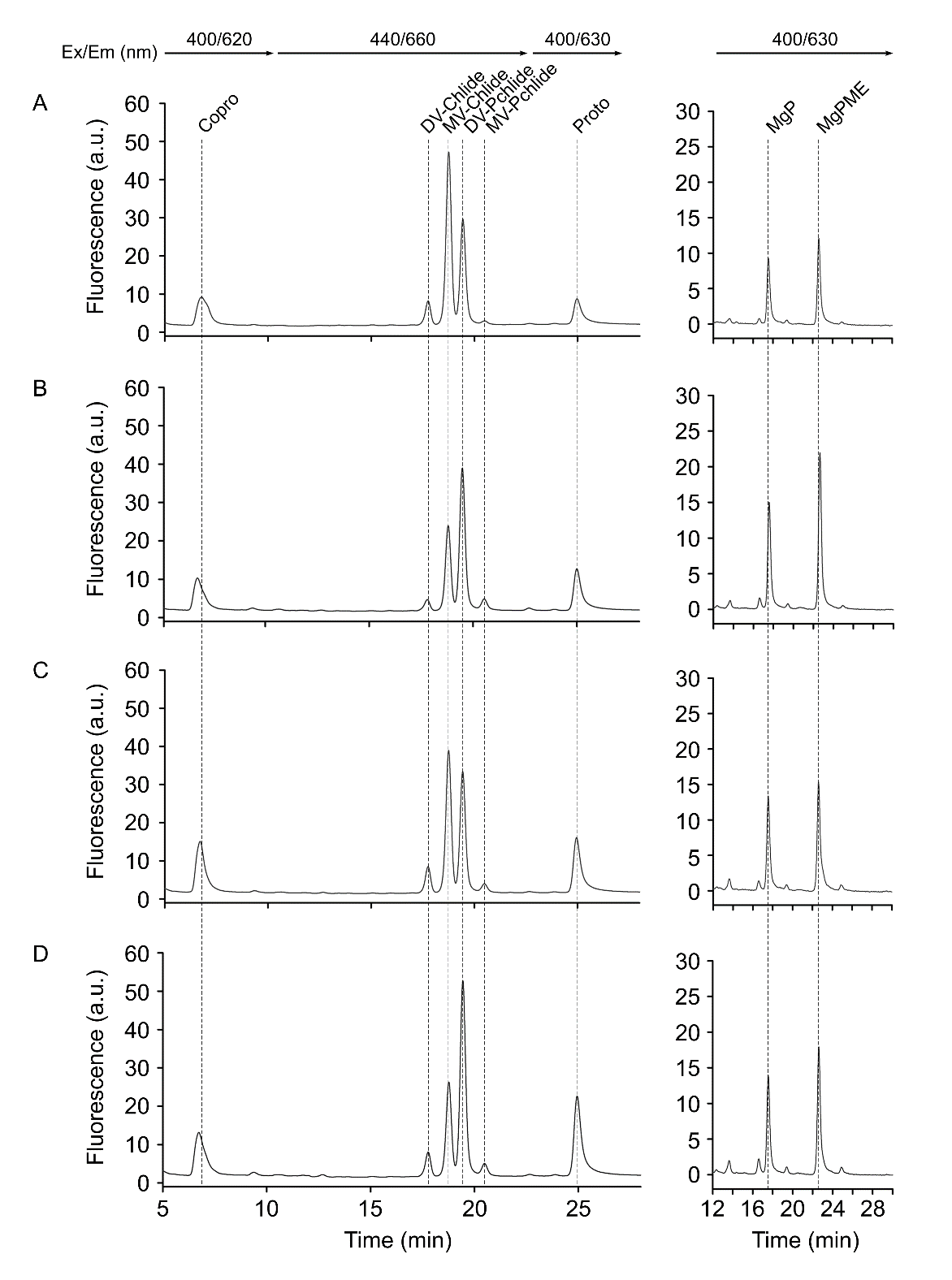
**

**Supplementary Fig. S2. Analysis of chlorophyll precursors from strains used in this study.** (A) FLAG-6803. (B) FLAG-7002. (C) FLAG-Cr. (D) FLAG-At. Pigments were extracted from cell pellets and separated by reverse phase HPLC. The left and right hand chromatograms were recorded simultaneously using two fluorescence detectors. The excitation (Ex) and emission (Em) wavelengths used are shown above the chromatograms. Copro = coproporphyrin IX; DV-Chlide = divinyl chlorophyllide *a*; MV-Chlide = monovinyl chlorophyllide *a*; DV-PChlide = divinyl protochlorophyllide *a*; MV-PChlide = monovinyl protochlorophyllide *a*; Proto = protoporphyrin IX; MgP = Mg-protoporphyrin IX; MgPME = Mg-protoporphyrin IX monomethyl ester.

**
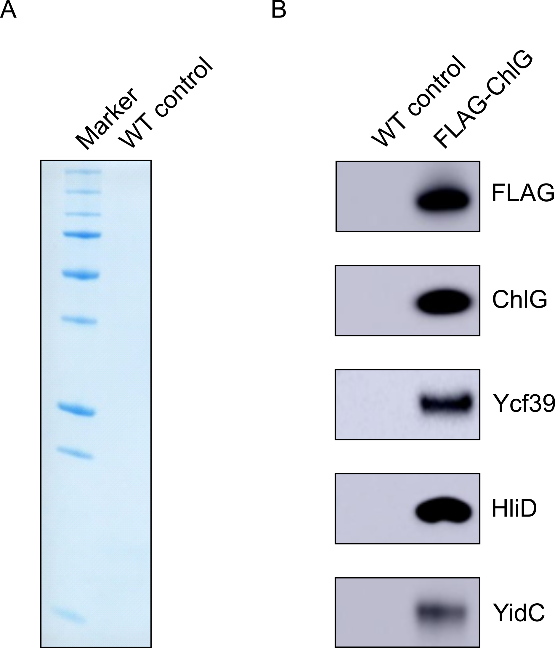
**

**Supplementary Fig. S3**. **SDS-PAGE and immunoblot analysis of control FLAG-immunoprecipitations from solubilized WT membranes.** Parallel immunoprecipitations were performed using solubilized membranes prepared from FLAG-6803 or WT *Synechocystis* cells. (A) SDS-PAGE analysis of WT elution. (B) Immunoblot analysis of WT versus FLAG-6803 eluates. None of the proteins identified in the FLAG-ChlG co-immunoprecipitation complex were detected in the WT control despite 20-fold higher loading, confirming that they do not non-specifically interact with the anti-FLAG resin.


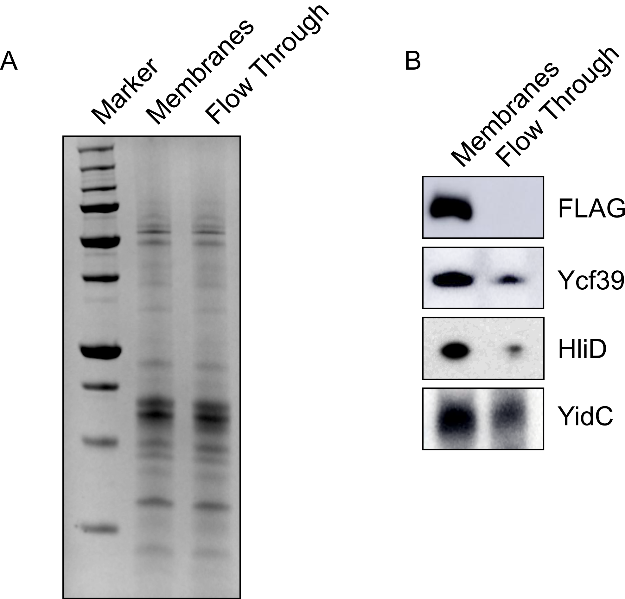


**Supplementary Fig. S4. Comparison of solubilized FLAG-6803 membranes applied to anti-FLAG resin versus the flow through that did not bind.** (A) Coomassie stained SDS-PAGE of membranes and flow through. (B) Immunodetection of FLAG-ChlG in the same fractions show all FLAG-tagged bait is captured by the column. Conversely partner proteins are all present in the flow through but in reduced levels compared to the membranes, consistent with a proportion of each being sequestered by FLAG-ChlG.

**
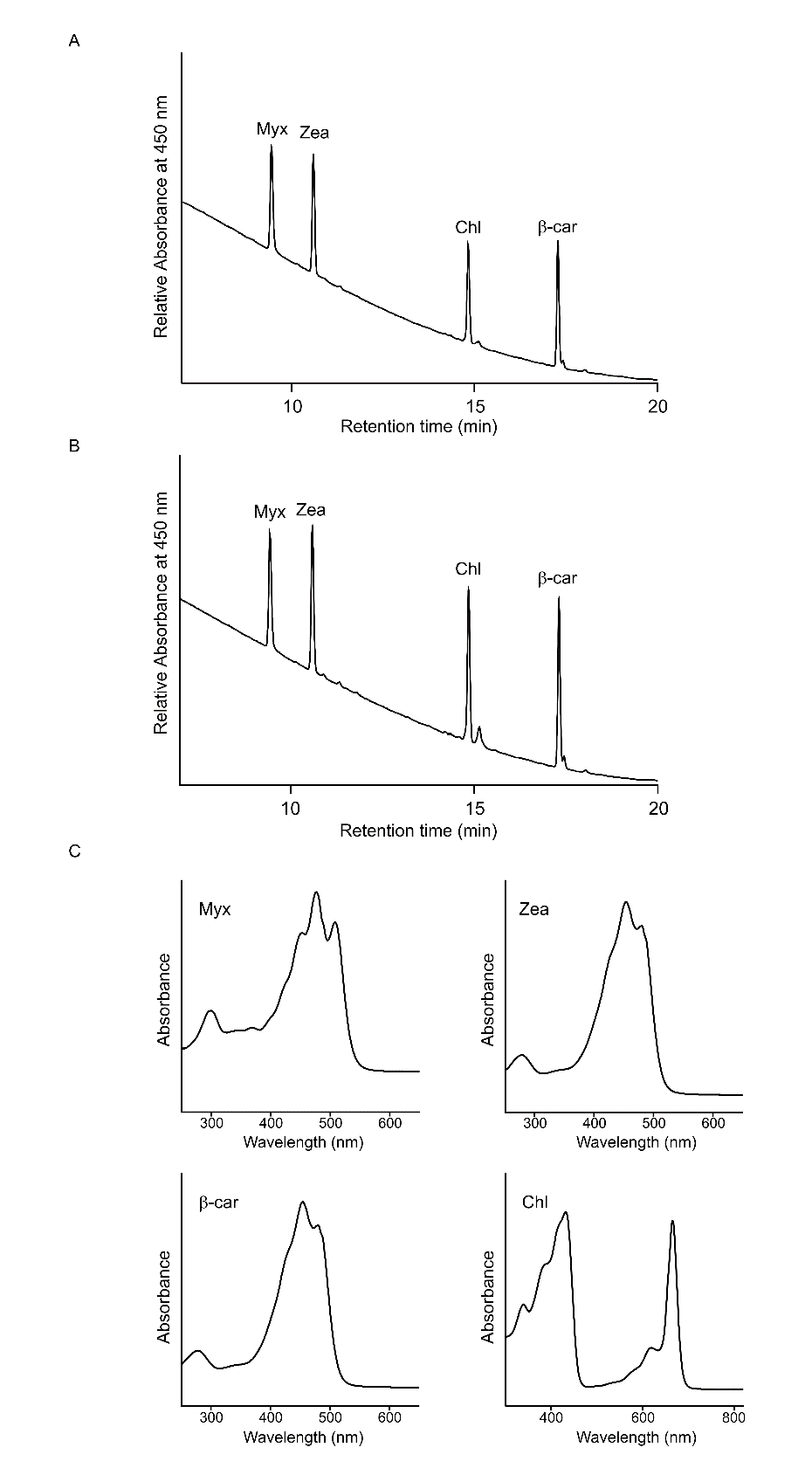
**

**Supplementary Fig. S5**. **Analysis of the pigment content of the cyanobacterial FLAG-ChlG co-immunoprecipitation eluates.** (A) FLAG-6803. (B) FLAG-7002. Pigments were extracted in methanol and separated by reverse phase HPLC, as described in the Materials and methods. The profiles were similar for both the cyanobacterial complexes, with myxoxanthophyll (Myx), zeaxanthin (Zea), β-carotene (β-car) and chlorophyll (Chl) all present. (C) Pigments were identified by their retention time and absorbance spectra.

**
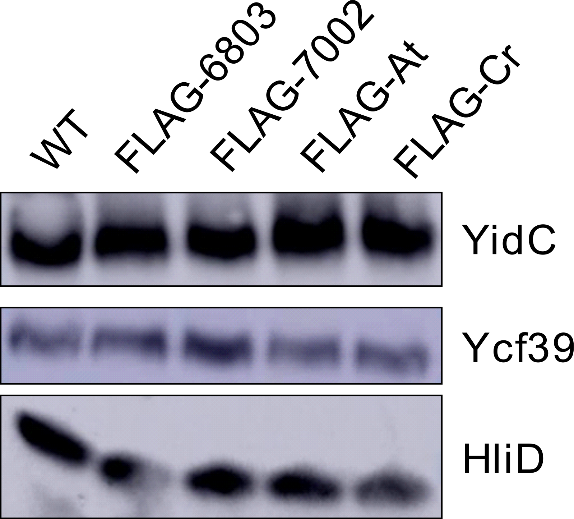
**

**Supplementary Fig. S6**. **Immunodetection of YidC, Ycf39 and HliD in solubiliszed membranes of different strains used in this study.** Membranes prepared from cells grown under standard illumination were probed with anti-YidC, anti-Ycf39 and anti-HliD, showing that the heterologous chlorophyll synthases do not have a significant effect on the cellular levels of these proteins.

**
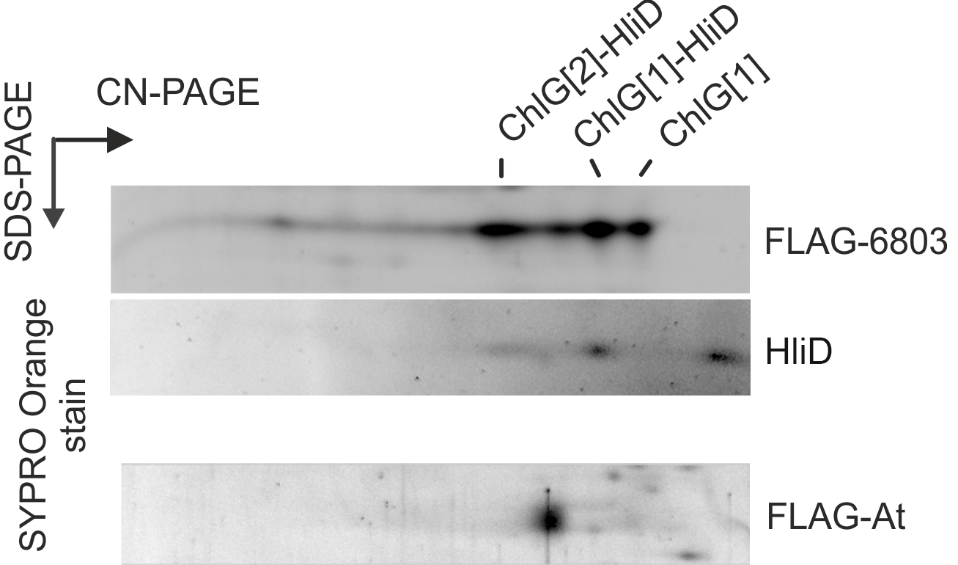
**

**Supplementary Fig. S7.** **Immunoprecipitation eluates from FLAG-6803 and FLAG-At strains separated by 2D CN/SDS-PAGE and stained by SYPRO Orange**. For FLAG-6803 a number of different sub-complexes of ChlG are present, as indicated above the gel; note that HliD has a tendency to dissociate from ChlG on this gel system [7]. Conversely the FLAG-At eluate has one major bind, which we predict to be a dimer of ChlG. The difference in the pattern of complexes between the two co-immunoprecipitation eluates is consistent with separation by gel filtration chromatography (Figure 3D).

**
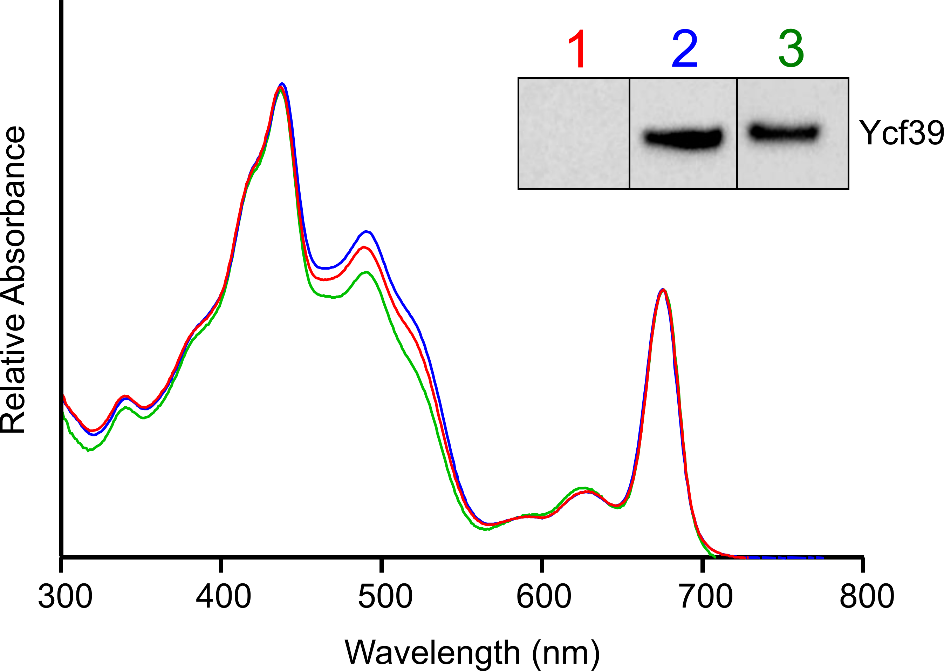
**

**Supplementary Fig. S8**. **Absorbance spectra of three independent FLAG-7002 ChlG immunoprecipitation eluates.** The inset panel shows immunodetection of Ycf39 in the same eluates.

**
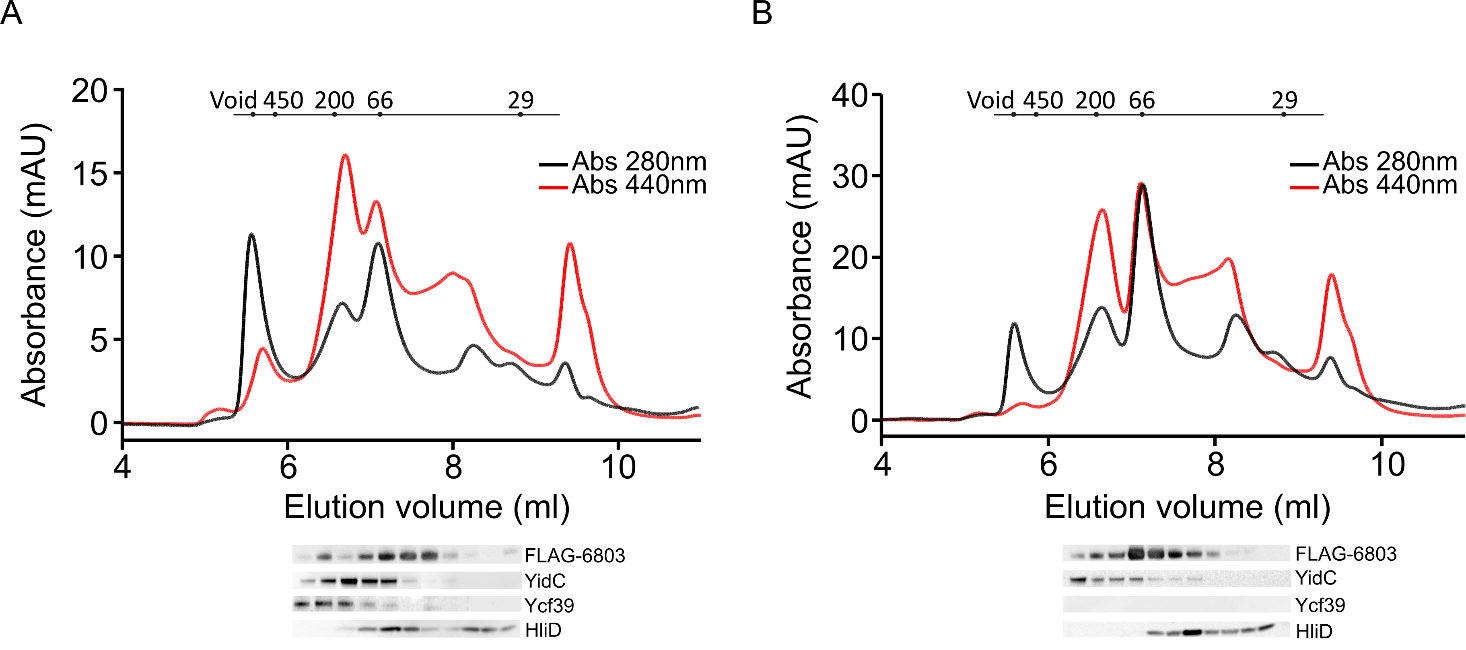
**

**Supplementary Fig. S9. Gel filtration chromatography of the pre and post light stress eluates.** (A) Pre light stress FLAG-ChlG co-immunoprecipitation eluate. (B) Post light stress FLAG-ChlG co-immunoprecipitation eluate. Protein and pigment were monitored by absorbance at 280 nm and 440 nm respectively. Immunoblot analysis of the corresponding 0.2 ml HPLC elution fractions are shown below the traces, confirming that Ycf39 is absent from the post light stress complex.


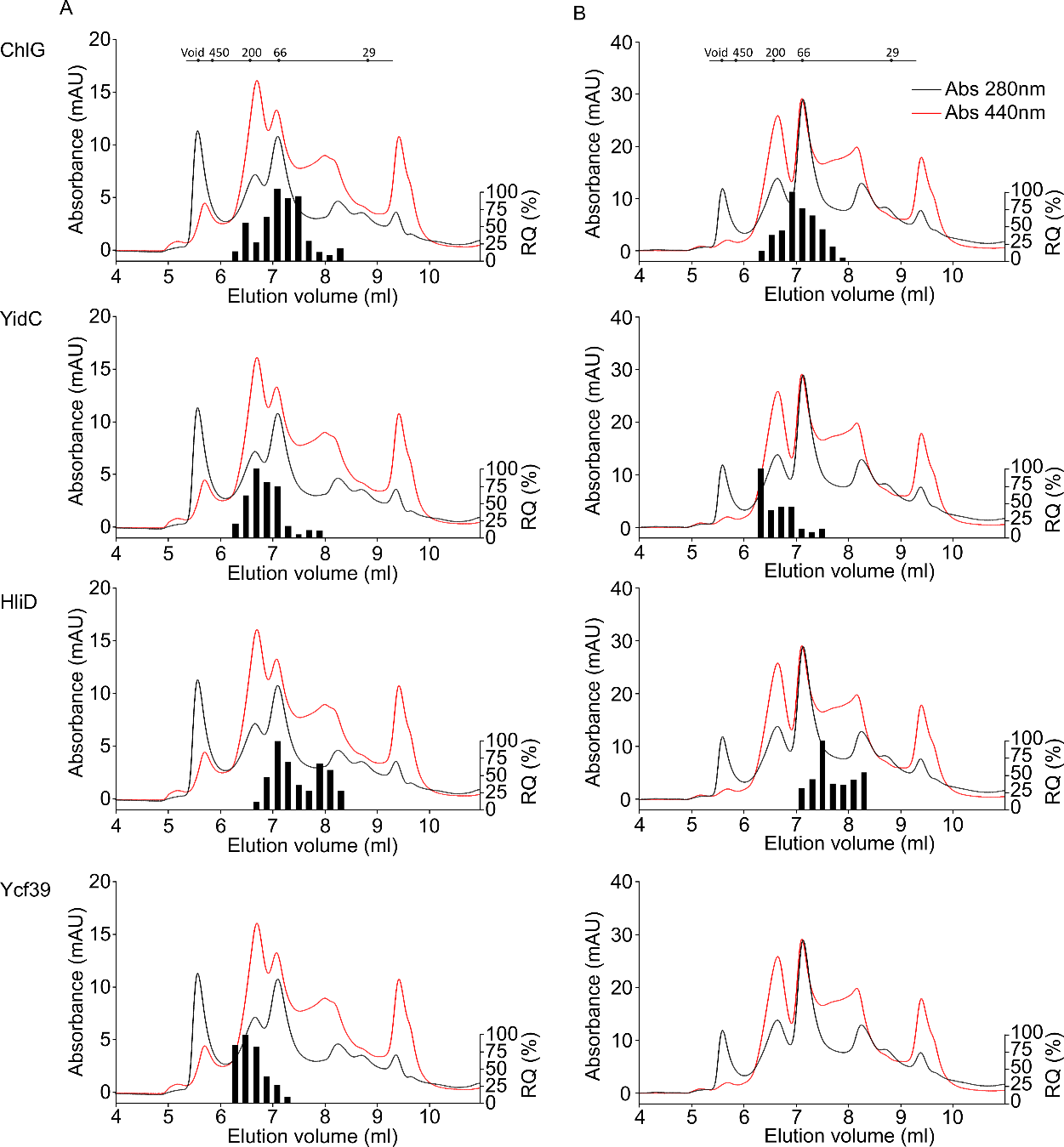


**Supplementary Fig. S10. Relative distribution of the components of the pre and post light stress eluates separated by gel filtration chromatography.** (A) Pre light stress FLAG-ChlG co-immunoprecipitation eluate. (B) Post light stress FLAG-ChlG co-immunoprecipitation eluate. The relative quantity (RQ) of each component was determined from the immunoblots in Fig. S7 and are presented as the percentage of the highest value in that blot. Each bar in the histogram represents the 0.2 ml elution fraction to which it is aligned.
